# Supplementary material for: Pre-Synaptic GABAA in NaV1.8+ Primary Afferents Is Required for the Development of Punctate but Not Dynamic Mechanical Allodynia following CFA Inflammation
Source: Cells. 2022 Aug 3;11(15):2390. doi: 10.3390/cells11152390 (PMC9368720; doi:10.3390/cells11152390)
Supplement: Supplementary file 1 [file cells-11-02390-s001.zip › cells-1805039-supplementary.pdf]

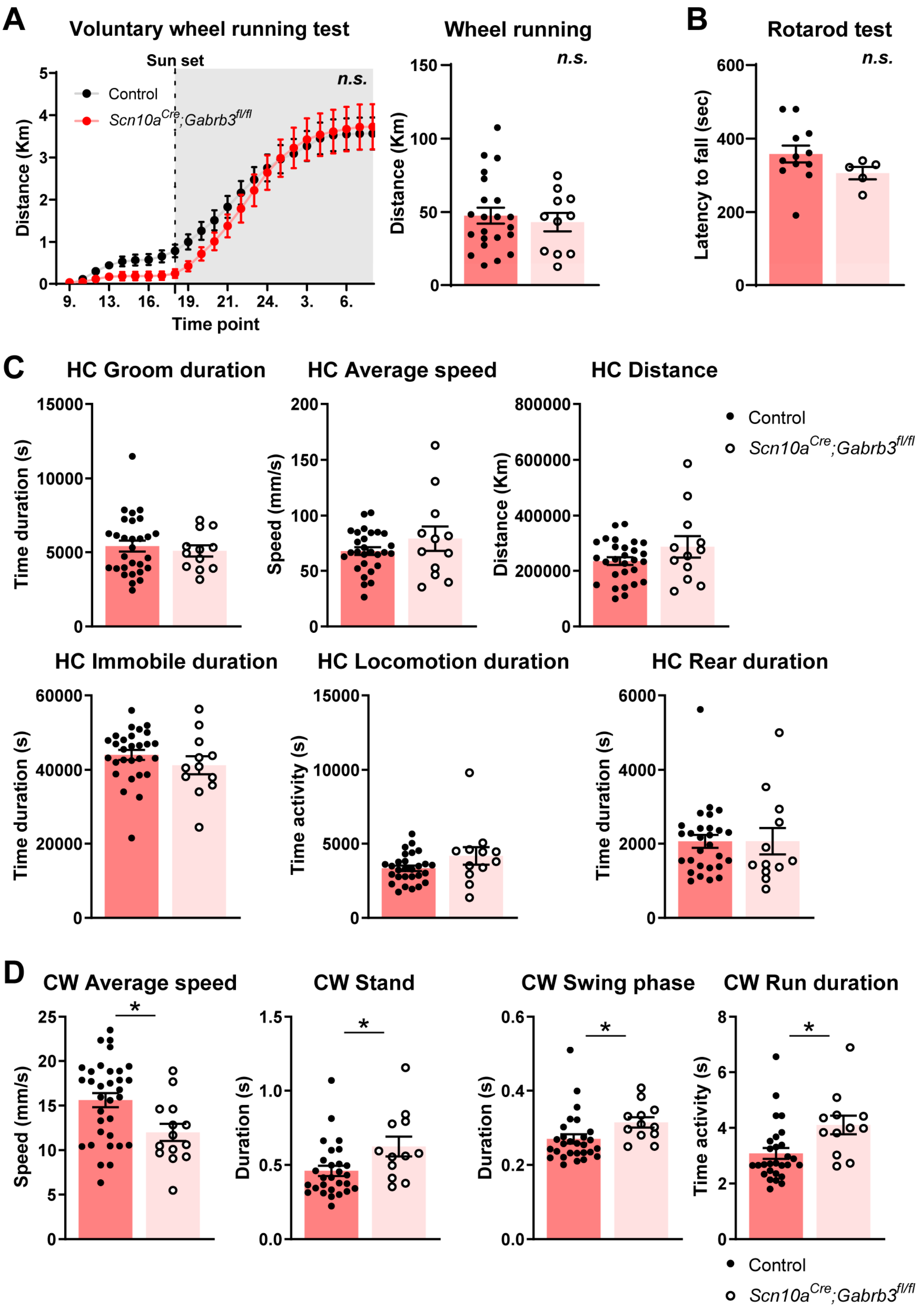

Figure S1: Phenotype for voluntary (A-B) and exploratory (C) motor behavior as well as locomotion (D) in Scn10a^{Cre};Gabrb3$^{fl/fl}$ (open markers and pink bars) and littermate controls (closed markers and red bars). A, Distance as a function of time of day of voluntary wheel running (left, A) and total cumulative distance within 24 hours (right, A). B, Latency (in seconds) from the time of placement onto a rotarod until the mouse fell off the rotarod. C, home cage (HC) activity over a 24 hours period. D, Cat walk (CW) automated gait analysis parameters for voluntary walking. Values of $p < 0.05$ were considered significant and are indicated with a asterisks as follows; * $p < 0.05$ and ** $p < 0.01$. "n.s." indicates not significant.

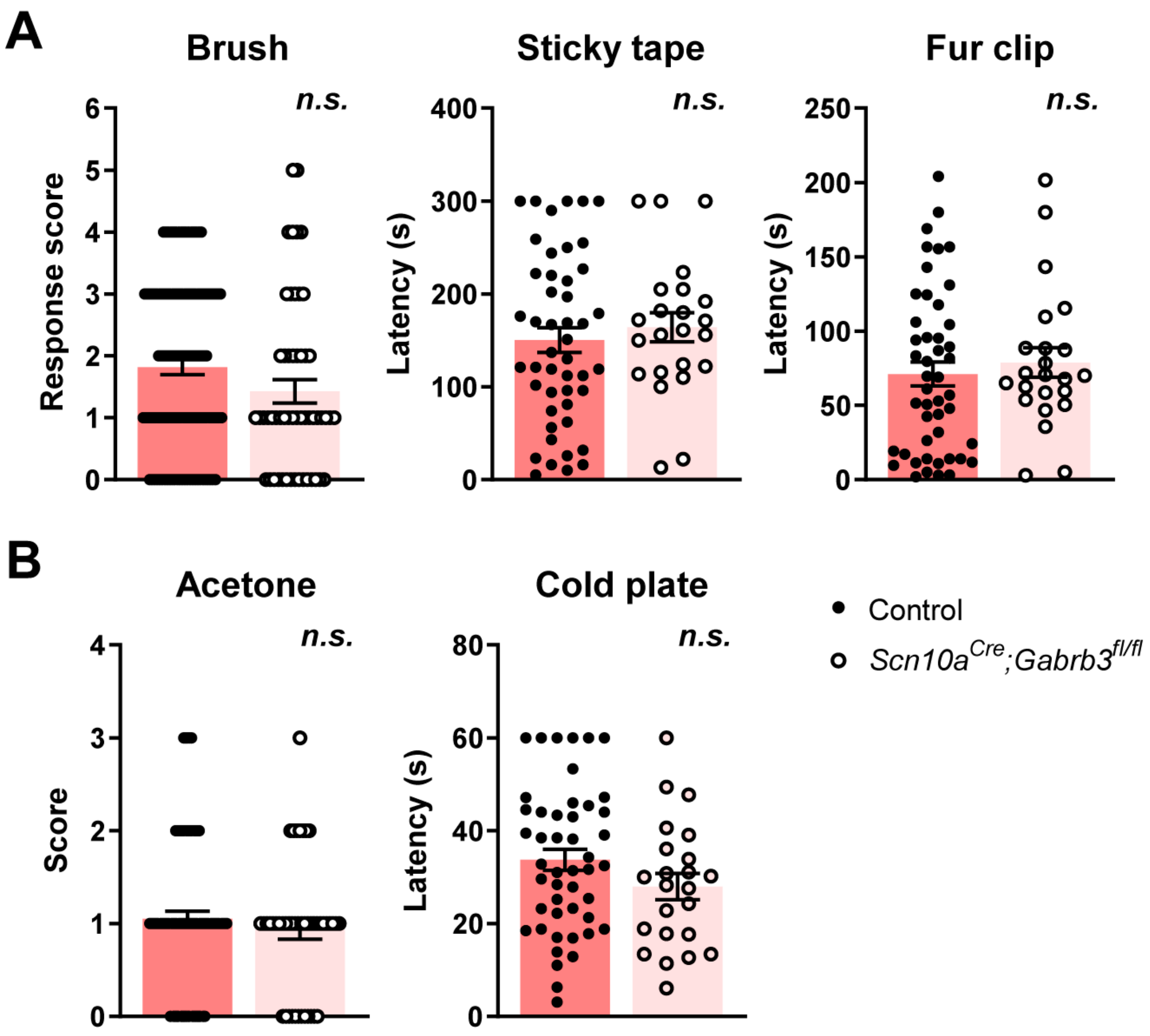

Figure S2: Phenotype for evoked sensory response in Scn10a^{Cre};Gabrb3$^{fl/fl}$ (open markers and pink bars) and littermate controls (closed markers and red bars). A, left, behavioral score of responses to stroking of the glabrous skin on the sole of the hindpaw with a paint brush. A, center and right latency (in seconds) to an overt attempt by the mouse to try to remove either sticky tape (centre, A) or a fur clip (right, A). B, left, behavioral score of responses to topical application of acetone as a cooling stimulus to the glabrous skin on the sole of the hindpaw. B, right, latency (in seconds) to paw withdrawal upon placement of the mouse onto a 5°C cold plate**.** "n.s." indicates not significant.

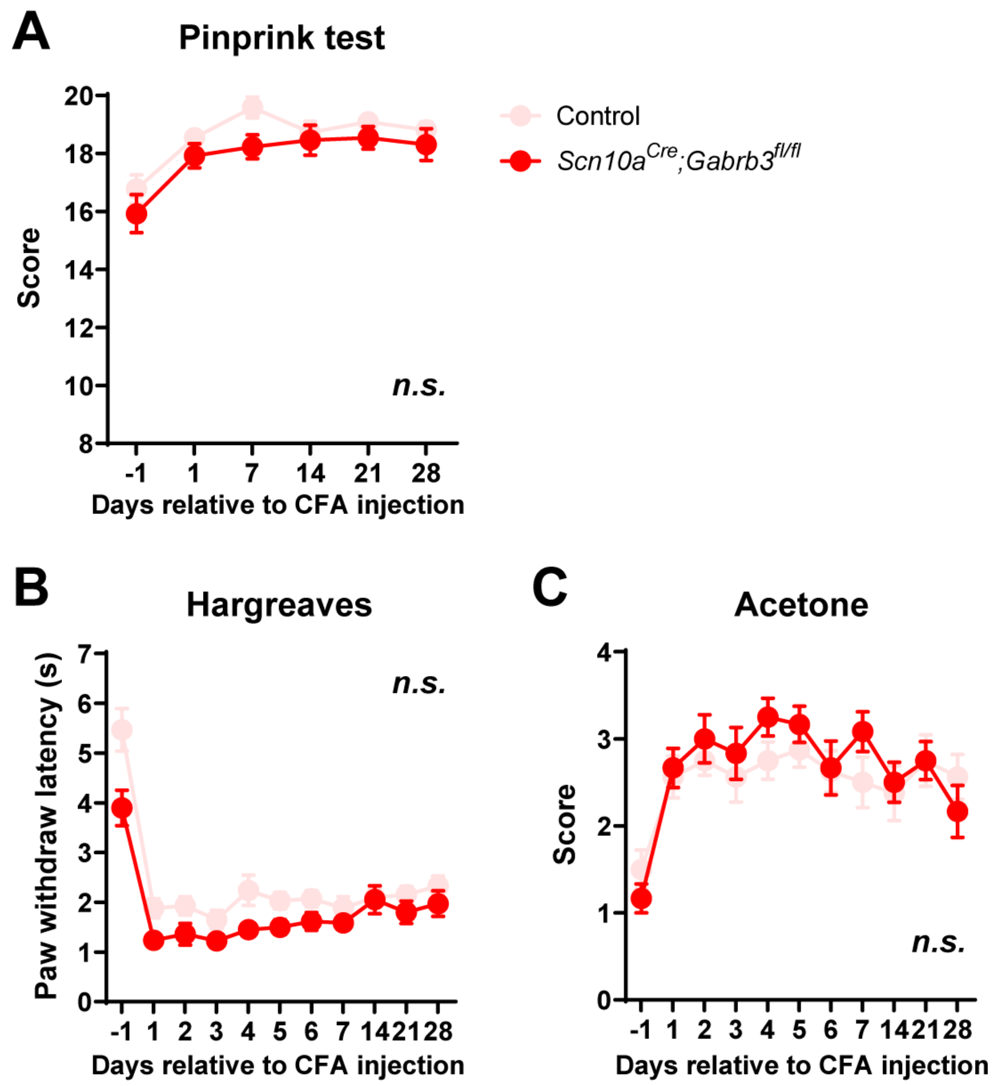

Figure S3: Development of sensory changes before and after CFA injection into the hindpaw of *Scn10a*^{Cre};*Gabrb3*$^{fl/fl}$ (red markers) and littermate control mice (pink markers). A, Behavioral score of responses to 1 gram pin prick stimulation before and after CFA injection. B, Time to paw withdrawal in seconds in response to radiant heat before and after CFA injection. C, Behavioral score of responses to acetone (cold sensitivity) before and after CFA injection. “n.s.” indicates not significant.

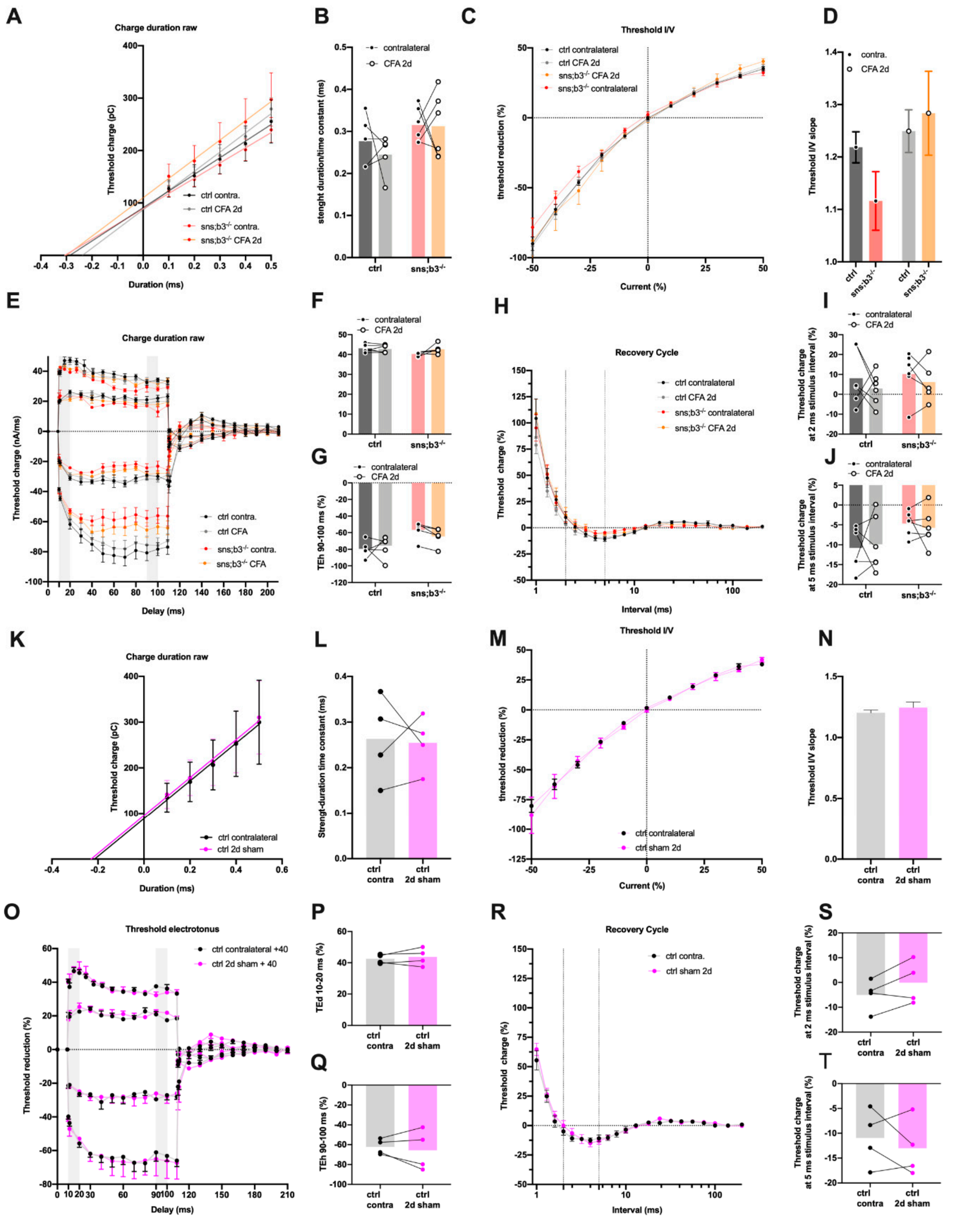

Figure S4: Multiple measures of excitability for myelinated A-fibre axons sural nerve. A-J, Comparisons between sural nerves from control mice (ctrl, black and grey bars) and *Scn10a*^{Cre};*Gabrb3*$^{fl/fl}$ mice (pink and orange bars). K-T, Comparisons between sural nerve responses taken from sham injected (ctrl sham, grey) and non-injected contralateral legs exclusively from control mice (ctrl contra, pink). Values of $p < 0.05$ were considered significant and are indicated with a asterisks as follows; * $p < 0.05$ and ** $p < 0.01$. "n.s." indicates not significant.
